# Sialic acid and PirB are not required for targeting of neural circuits by neurotropic mammalian orthoreovirus

**DOI:** 10.1128/msphere.00629-24

**Published:** 2024-09-25

**Authors:** Kira A. Griswold, Iaroslavna Vasylieva, Megan C. Smith, Kay L. Fiske, Olivia L. Welsh, Alexa N. Roth, Alan M. Watson, Simon C. Watkins, Danica M. Sutherland, Terence S. Dermody

**Affiliations:** 1 Department of Microbiology and Molecular Genetics, University of Pittsburgh School of Medicine, Pittsburgh, Pennsylvania, USA; 2 Institute of Infection, Inflammation, and Immunity, UPMC Children’s Hospital of Pittsburgh, Pittsburgh, Pennsylvania, USA; 3 Department of Cell Biology, University of Pittsburgh, Pittsburgh, Pennsylvania, USA; 4 Center for Biologic Imaging, University of Pittsburgh, Pittsburgh, Pennsylvania, USA; 5 Department of Pediatrics, University of Pittsburgh School of Medicine, Pittsburgh, Pennsylvania, USA; Instituto de Biotecnologia/UNAM, Cuernavaca, Morelos, Mexico

**Keywords:** reovirus, neurotropic viruses, neuropathogenesis, CLARITY, MiPACT-HCR, PirB, sialic acid

## Abstract

Serotype 3 (T3) strains of mammalian orthoreovirus (reovirus) spread to the central nervous system to infect the brain and cause lethal encephalitis in newborn mice. Although reovirus targets several regions in the brain, susceptibility to infection is not uniformly distributed. The neuronal subtypes and anatomic sites targeted throughout the brain are not precisely known. Reovirus binds several attachment factors and entry receptors, including sialic acid (SA)-containing glycans and paired immunoglobulin-like receptor B (PirB). While these receptors are not required for infection of some types of neurons, reovirus engagement of these receptors can influence neuronal infection in certain contexts. To identify patterns of T3 neurotropism, we used microbial identification after passive tissue clearance and hybridization chain reaction to stain reovirus-infected cells throughout intact, optically transparent brains of newborn mice. Three-dimensional reconstructions revealed in detail the sites targeted by reovirus throughout the brain volume, including dense infection of the midbrain and hindbrain. Using reovirus mutants incapable of binding SA and mice lacking PirB expression, we found that neither SA nor PirB is required for the infection of various brain regions. However, SA may confer minor differences in infection that vary by region. Collectively, these studies indicate that many regions in the brain of newborn mice are susceptible to reovirus and that patterns of reovirus infection are not dependent on reovirus receptors SA and PirB.

**IMPORTANCE:**

Neurotropic viruses invade the central nervous system (CNS) and target various cell types to cause disease manifestations, such as meningitis, myelitis, or encephalitis. Infections of the CNS are often difficult to treat and can lead to lasting sequelae or death. Mammalian orthoreovirus (reovirus) causes age-dependent lethal encephalitis in many young mammals. Reovirus infects neurons in several different regions of the brain. However, the complete pattern of CNS infection is not understood. We found that reovirus targets almost all regions of the brain and that patterns of tropism are not dependent on receptors sialic acid and paired immunoglobulin-like receptor B. These studies confirm that two known reovirus receptors do not completely explain the cell types infected in brain tissue and establish strategies that can be used to understand complete patterns of viral tropism in an intact brain.

## INTRODUCTION

Neurotropic viruses infect the nervous system and can cause life-threatening disease. Differences in cell tropism of neurotropic viruses result in varying disease manifestations, such as meningitis, myelitis, and encephalitis (1). Following invasion of the central nervous system (CNS), neurotropic viruses can target endothelial cells, diverse neuronal subtypes in the brain or spinal cord, or resident immune cells such as microglia (1). Tropism differences can be dictated by the route of dissemination, as is the case with West Nile virus (2), expression of viral receptors, as is the case with rabies virus (3), or a variety of other cell-intrinsic factors. Understanding mechanisms of viral tropism in the nervous system can provide clues about the host factors that dictate cell susceptibility to infection, which in turn might yield therapeutic targets.

Mammalian orthoreovirus (reovirus) assembles nonenveloped, double-shelled virions that package 10 segments of double-stranded RNA encoding 12 proteins (4). Following inoculation into the intestine of naive, newborn mice, reovirus infects robustly, establishes viremia, and spreads to a variety of tissues, including the heart, liver, and brain (4). Reovirus causes serotype-specific disease in the CNS, wherein serotype 1 (T1) reovirus infects ependymal cells and causes non-lethal hydrocephalus (5), and serotype 3 (T3) reovirus infects neurons and causes lethal encephalitis (6, 7). T3 reovirus spreads from sites of primary infection to the brain using both hematogenous and neural routes (8). The reovirus σ1 attachment protein, which is encoded by the S1 gene segment, dictates viral tropism for neurons (6, 7, 9). The basis for reovirus serotype-dependent differences in neural tropism is not understood, but T1 and T3 reoviruses are thought to engage different, as-yet-unidentified receptors expressed on ependymal cells and neurons, respectively (10). Thus, while viral determinants of neuropathogenesis have been identified, the underlying host factors responsible for reovirus neurotropism are less clear.

Reovirus targets several regions in the brain, including the cortex, hippocampus, and cerebellum. However, within these regions, susceptibility to reovirus infection is not uniformly distributed (6, 7, 9, 11, 12). Instead, reovirus tropism is highly specific for different neuronal subtypes. For example, reovirus infects neurons in cortical layers IV and V, hippocampal regions CA2 and CA3, and Purkinje neurons in the cerebellum, whereas neighboring neurons are relatively spared (6, 7, 9, 11, 12). However, a complete description of the neuronal subtypes and regions targeted throughout the brain has not been documented. Many studies have focused on the identification of factors governing reovirus tropism by evaluating patterns of infection in mice following inoculation with different T3 strains at varying doses and inoculation routes and the use of host species with different genetic backgrounds. No major differences in reovirus tropism have been described by altering any of these conditions (9, 11, 13, 14). Additionally, it is not clear whether identified reovirus attachment factors or receptors expressed in the CNS contribute to viral tropism in the nervous system.

Reovirus binds several types of attachment factors and receptors that in concert lead to virus uptake into cells. The reovirus capsid incorporates three proteins that interact with attachment factors or receptors in mice, viral attachment protein σ1 (15, 16), outer-capsid protein σ3 (13), and core protein λ2 (17). Sialic acids (SAs) are attachment factors bound by reovirus σ1 protein in a serotype-specific manner to promote viral attachment to cells (18–21). T3 σ1 engages a variety of sialylated glycans (19) and, while SA distribution varies by cell and tissue type, all normal vertebrate cells express SAs. A reovirus mutant incapable of interacting with SAs has diminished neurovirulence (14). However, the influence of SA engagement on reovirus neurovirulence could be related to dose, route of entry, or genetic background of the host species (14). Reovirus capsid protein σ3 binds paired immunoglobulin-like receptor B (PirB) (13), which is expressed on immune cells (22) and neurons (23). In mice lacking PirB, reovirus loads are diminished at early time points, and reovirus virulence is decreased, indicating that PirB contributes to reovirus neuropathogenesis. However, PirB expression does not completely explain neural patterns of T3 reovirus infection, as neural-specific PirB^-/-^ mice do not appear to restrict reovirus infection in the brain, and these animals remain susceptible to reovirus-induced lethal encephalitis (13). It is not known whether sialylated glycans or PirB influence patterns of reovirus tropism in the CNS. Insights into viral neurotropism have improved an understanding of viral transmission and disease outcomes. For example, rabies virus infection of nicotinic acetylcholine receptor-expressing neurons in the hippocampus is thought to contribute to hyperactivity and aggression in affected mammals (24). Thus, examination of viral tropism in the CNS can provide clues to the basis of disease manifestations.

In this study, we used a hybridization chain reaction (HCR) approach to identify reovirus-infected cells in both whole brain tissue and histological brain slices. We established new techniques to evaluate reovirus tropism in intact tissue using whole-organ clearing and HCR and quantified three-dimensional (3D) data sets by aligning infected mouse brains with publicly available brain atlases. We detected reovirus-susceptible cells distributed throughout most regions of the brain imaged and found the highest density of infection in the hindbrain. Using genetically altered viruses and mice, we found that engagement of sialylated glycans or PirB is not required for patterns of reovirus neurotropism in the brain. These results indicate that host factors other than SA and PirB influence reovirus susceptibility in the CNS and establish new strategies to assess differences in viral tropism.

## RESULTS

### Hybridization chain reaction detects reovirus RNA

**Fig 1:**
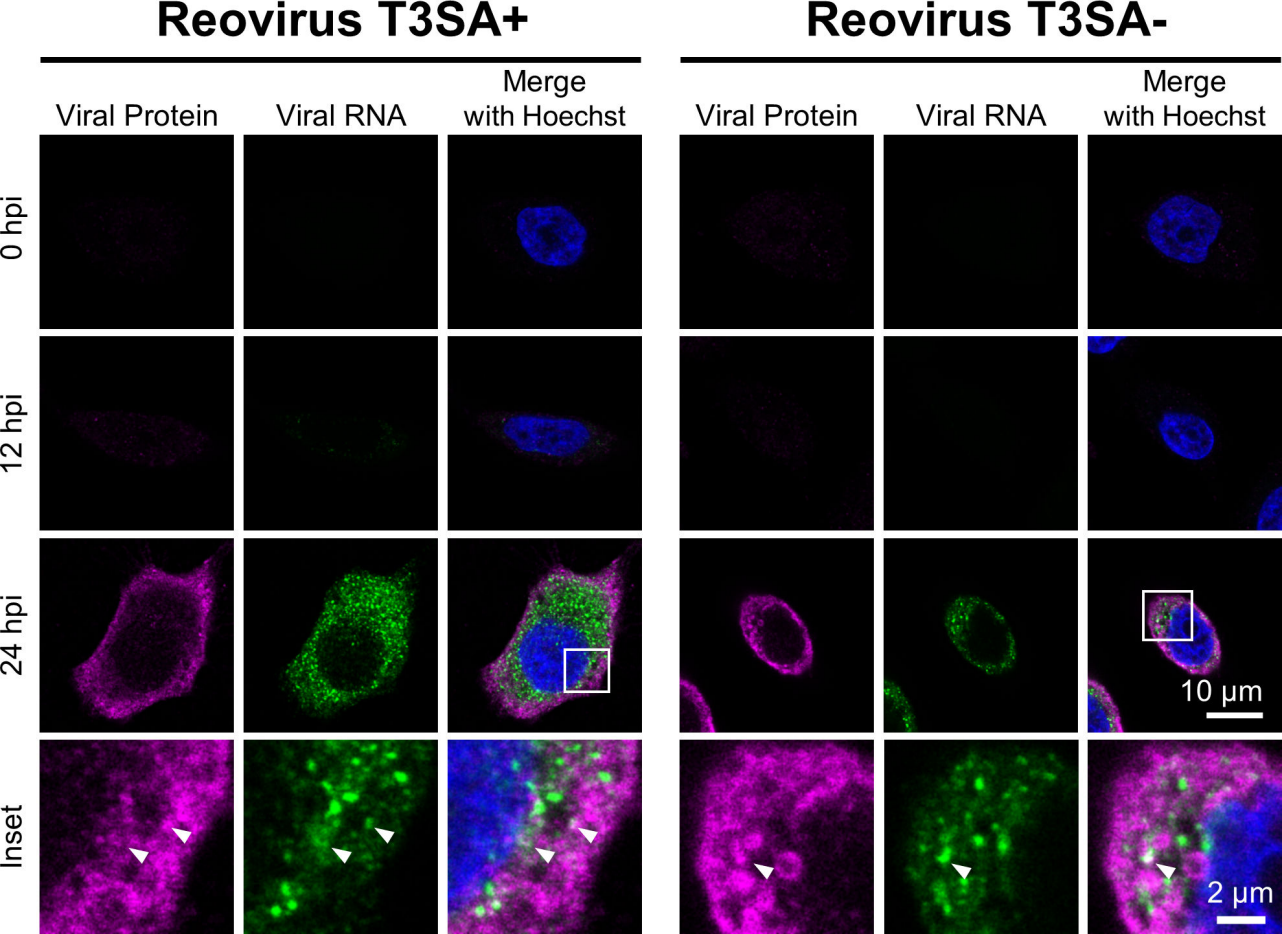
HCR assay detects reovirus RNA at late stages of infection in cultured cells. HeLa cells were adsorbed with 100 PFU/cell of reovirus strains T3SA+ or T3SA− for 1 h. At the times post-adsorption shown, cells were fixed and stained for reovirus S3 RNA (green) by HCR and viral protein (magenta) by indirect immunofluorescence. Cells were counterstained with Hoechst dye (blue) and imaged using confocal microscopy. Representative images are shown. Arrowheads indicate sites of colocalization. Scale bar, 10 µm (single cell) or 2 µm (inset).

To assess reovirus neurotropism in intact brain tissue, we first determined whether the detection of viral RNA would provide a sensitive and specific approach. Advances in RNA fluorescence *in situ* hybridization (FISH) technologies, including HCR, allow the detection of specific RNAs and amplification of the resultant signal (25). We designed 10 DNA initiator HCR probe pairs to detect reovirus S3 RNA (26) (Table 1). For all experiments in this study, we used reovirus strains T3SA+ (glycan-binding) and T3SA− (glycan-blind), which differ by a single amino acid polymorphism in σ1 that dictates SA binding (18). To validate the probes, HeLa cells were adsorbed with T3SA+ or T3SA−, fixed at various times post-adsorption, and stained for reovirus RNA by HCR and reovirus protein by indirect immunofluorescence assay (Fig. 1). At 24-h post-adsorption, viral protein and RNA were detected in infected cells, with some colocalization of signals observed at sites of viral factories. Although the use of HCR staining reagents may minimize antigen detection, these data establish HCR as a reliable strategy to identify cells with actively replicating reovirus.

**TABLE 1:** Virus-specific HCR probes^*a*^

Probe pair name	Complementary to hairpin–spacer–*complementary to RNA of interest*
T1L-S3-P1a	**GAGGAGGGCAGCAAACGG–**AA**–***TGAGTGAGGAAGCCATAGTGACGAC*
T1L-S3-P1b	*CTCTCTTGATCTTAGAGATCGCAGC***–**TA**–GAAGAGTCTTCCTTTACG**
T1L-S3-P2a	**GAGGAGGGCAGCAAACGG–**AA**–***TCACGTTTAGCATACTGGAGCGGTC*
T1L-S3-P2b	*TCCAGATTCCTTTGTCCAAGCAGAC***–**TA**–GAAGAGTCTTCCTTTACG**
T1L-S3-P3a	**GAGGAGGGCAGCAAACGG–**AA**–***TAGCTGCAGATCAGCTCTCTCATGG*
T1L-S3-P3b	*AACCCATCAGTGTGGTCGACCTTGA***–**TA**–GAAGAGTCTTCCTTTACG**
T1L-S3-P4a	**GAGGAGGGCAGCAAACGG–**AA**–***CCATACCCATAGTGAACAATCTGGC*
T1L-S3-P4b	*AAGGCTCAGTGGTAATGTGTAGTCC***–**TA**–GAAGAGTCTTCCTTTACG**
T1L-S3-P5a	**GAGGAGGGCAGCAAACGG–**AA**–***AAGCTAACGTCATGCAGTCCAAATC*
T1L-S3-P5b	*CACCATCAAGTGTAATCATGTAGGG***–**TA**–GAAGAGTCTTCCTTTACG**
T1L-S3-P6a	**GAGGAGGGCAGCAAACGG–**AA**–***GCTAATCCCTTAAGTCCATCATCC*
T1L-S3-P6b	*TCACATCCGTATGAGATATCCATGC***–**TA**–GAAGAGTCTTCCTTTACG**
T1L-S3-P7a	**GAGGAGGGCAGCAAACGG–**AA**–***TTGATGCATCGTGAAGAATCCATGC*
T1L-S3-P7b	*CTGCCGTTTCCTCGCAATATAACT***–**TA**–GAAGAGTCTTCCTTTACG**
T1L-S3-P8a	**GAGGAGGGCAGCAAACGG–**AA**–***ATCAATGGTAGCAAATGAAGAAGCC*
T1L-S3-P8b	*CATCACACAAAACCTGAAACACTGG***–**TA**–GAAGAGTCTTCCTTTACG**
T1L-S3-P9a	**GAGGAGGGCAGCAAACGG–**AA**–***AGTCCAAAGTCTCATCGTTTCACGC*
T1L-S3-P9b	*AACCACTGAATCGTCCAACGCCGAT***–**TA**–GAAGAGTCTTCCTTTACG**
T1L-S3-P10a	**GAGGAGGGCAGCAAACGG–**AA**–***TCGACCTCGATCCAGCGAAATACTC*
T1L-S3-P10b	*AGCATCAGTAGCATCAGCTGCTACC***–**TA**–GAAGAGTCTTCCTTTACG**

^*a*^ Sequences for HCR probes specific for the reovirus S3 gene. Bold sequences indicate complementarity to the hairpin; italic sequences indicate complementarity to the RNA of interest.

### Reovirus tropism is detected throughout the brain using MiPACT-HCR-SWITCH

**Fig 2:**
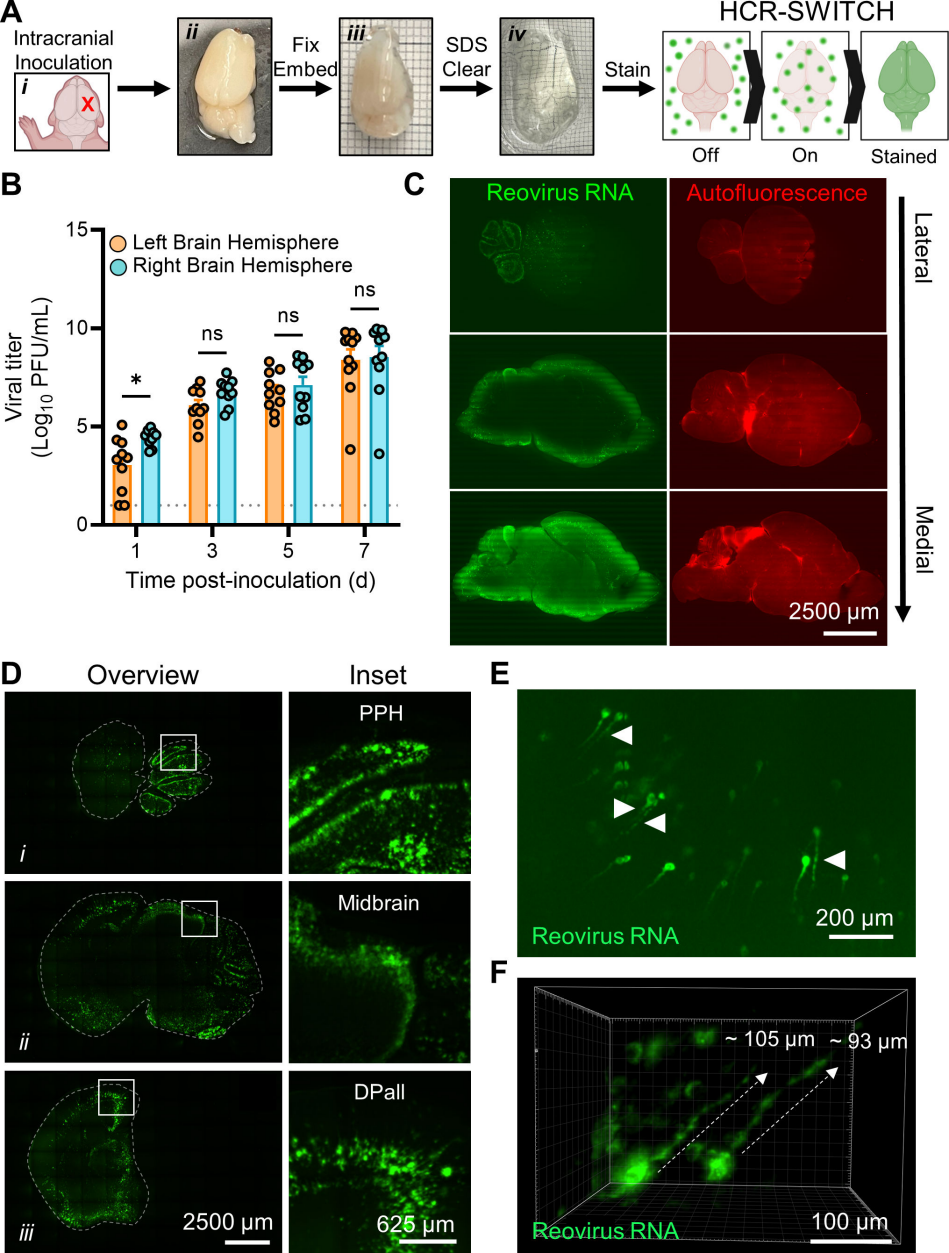
Viral replication sites are detected throughout the brain in cleared, reovirus-infected brain hemispheres. (**A–F**) Two-day-old WT mice were inoculated IC with phosphate-buffered saline (mock) or 1,000 PFU of T3SA+. Mice were euthanized at 7 days post-inoculation, and brains were resected and hemisected along the latitudinal fissure. (**A**) Schematic of the approach to stain whole, cleared tissues using MiPACT, SWITCH, and HCR (MiPACT-SWITCH-HCR). The inoculation site is indicated with an X (i). Right-brain hemispheres were fixed with paraformaldehyde (ii), embedded in an acrylamide-based gel (iii), cleared using SDS (iv), and processed for the detection of viral RNA using the SWITCH-HCR approach. Minor gridlines in (iii) and (iv), 1 mm. Tissue in (iv) is partially immersed in refractive index-matching solution (RIMS). (**B**) Titers of the virus in homogenized hemispheres (right or left) were determined by plaque assay. Each symbol represents the viral titer from an individual animal. *N* = 10-11. Error bars indicate SEM. Values that differ significantly from the left-brain hemisphere by the mixed-effects model are indicated (*P < 0.05). *ns*, not significant. Dotted line indicates limit of detection. (**C**) Cleared, stained, and RIMS-submerged brain tissue was imaged in 3D using ribbon-scanning confocal microscopy. Data were processed using Imaris software (Oxford Instruments). Representative sagittal optical planes from a 3D-imaged brain data set depicting autofluorescence (right images, red) as a proxy for effective brain clearance or viral RNA signal (left images, green). Optical planes proceed from lateral (top) to medial (bottom) regions of the brain. Scale bars, 2,500 µm. (**D**) Representative sagittal optical planes from the 3D data set in panel (**C**) demonstrating reovirus-infected cells in different brain regions (i–iii). Left, brain (outlined by dashed line); right, enlarged inset boxed from the left image. Scale bars, 2,500 (whole-brain images) or 625 µm (insets). PPH, prepontine hindbrain; DPall, dorsal pallium. (**E and F**) Representative images from the 3D data set in panel (**C**) demonstrating reovirus-infected neurons in the brain. (**E**) Sagittal optical plane demonstrating reovirus RNA staining in viral factory-like patterns (arrowheads, globular structures). Scale bar, 200 µm. (**F**) Max projection reovirus RNA staining within axon-like extensions (dashed lines indicate approximate length). Scale bar, 100 µm.

Previous studies using two-dimensional (2D) immunohistochemistry (IHC) to evaluate reovirus tropism in single brain-tissue slices (6, 7, 9, 11, 12) provide limited information about infection in the entire organ. To evaluate reovirus tropism in the intact brain, we developed a strategy to detect reovirus infection in optically cleared brain hemispheres. Two-day-old wild-type (WT) mice were inoculated intracranially (IC) with reovirus T3SA+ and euthanized at 7 days post-inoculation when peak viral loads would be anticipated and just prior to the onset of disease signs. Brain tissue was resected and hemisected, and tissue was fixed and embedded in bis-acrylamide to preserve tissue structure (Fig. 2A). Following embedding, light-absorbing lipids were removed from the brain using microbial identification after passive CLARITY technique (MiPACT) (27). Completely cleared tissues were stained using HCR paired with SWITCH (system-wide control of interaction time and kinetics of chemicals) (28) to mark infected cell foci throughout the brain volume (Fig. 2A). To determine the appropriate hemisphere to evaluate reovirus tropism, we assessed viral load in both the inoculated and the contralateral hemisphere and found that there was no significant difference in viral load in either brain hemisphere at late stages of disease (Fig. 2B). We therefore proceeded with imaging the inoculated right-brain hemisphere. Cleared brains were imaged using either ribbon-scanning confocal microscopy (29) or mesoscale selective-plane illumination microscopy (MesoSPIM) (30) (Fig. 2C through F). Ribbon-scanning confocal microscopy was developed to process 3D images at higher speeds than traditional confocal microscopy (29), whereas the MesoSPIM strategy uses high-speed light-sheet imaging to minimize image distortion that occurs during volumetric confocal imaging (30). Both techniques can capture 3D information of stained tissue at comparable resolutions. A representative 3D rendering of stained brain tissue is shown as a movie (Movie S1A).

Brain architecture was captured using autofluorescence in the GFP channel, and staining of reovirus RNA was detected from the most medial (inner edge of the hemisphere) to the most lateral (outer edge of the hemisphere) sagittal planes (Fig. 2C). Many regions were infected with reovirus, with strong staining in the prepontine hindbrain, midbrain, and dorsal pallium (Fig. 2D). These data indicate that reovirus tropism is not restricted to a specific brain region and can be detected throughout the brain volume, consistent with previous studies of reovirus tropism using conventional IHC (6, 7, 9, 11, 12). Reovirus-infected cells were morphologically consistent with neurons (Fig. 2E and F). Viral RNA was evident in axon-like projections, and staining patterns consistent with viral factories were observed in these cell structures (Fig. 2E). Reconstructions in three dimensions revealed reovirus staining along approximately 100 µm of axon-like structures (Fig. 2F). Most viral RNA staining was localized to cell bodies. A representative 3D rendering of viral RNA detection in cells with neuron-like morphology is shown as a movie (Movie S1B).

### Alignment of reovirus-infected 3D brain to a developing mouse brain atlas reveals most subregions of the brain are susceptible to infection

**Fig 3:**
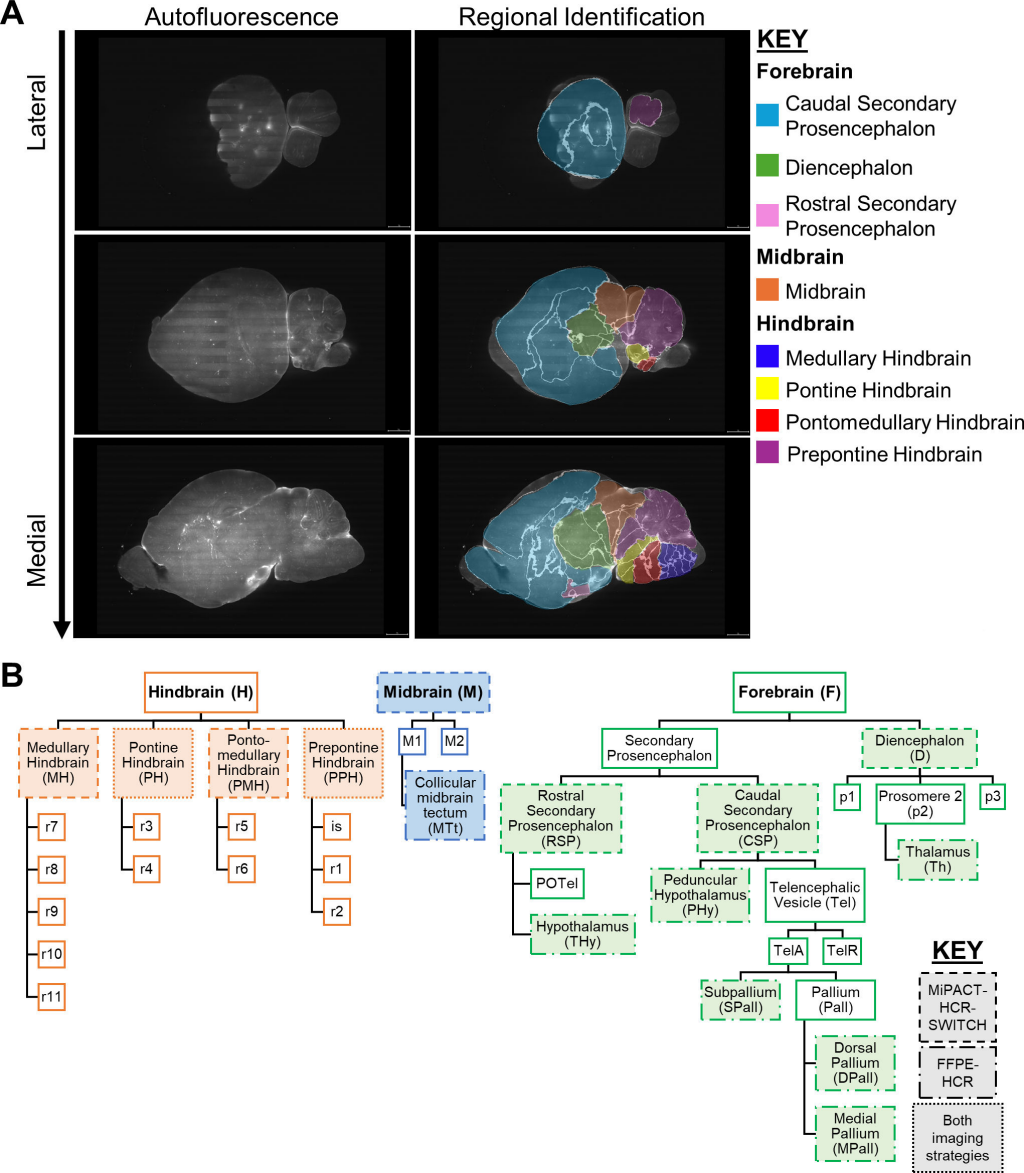
Schematics detailing developing mouse brain atlas overlay and regional segmentation of cleared brains. (**A and B**) The 3D-reconstructed Allen Developing Mouse Brain Reference Atlas (P14) (31–33) was aligned and overlaid with reovirus-infected brain data sets using the *brainreg* Python package. Allen Developing Mouse Brain Reference Atlas, https://developingmouse.brain-map.org. (**A**) Representative sagittal optical plane images depicting autofluorescence from brain tissue (left column) alone or overlaid with the fitted model from the 3D-reconstructed Allen Developing Mouse Brain Atlas—P14 (right column) (31–33). Brain subregions are colored according to identity; see key for details. (**B**) Schematic describing relationships between brain regions and subregions used for imaging studies; see key for details.

Successful imaging and reconstruction of reovirus-infected brain tissue identified several notable features of viral tropism. To systematically define the distribution of viral infection in brain tissue, we used the *brainreg* Python-based tool to automate the alignment of the 3D data sets from 9-day-old infected mice with the 14-day-old 3D-reconstructed Allen Developing Mouse Brain Atlas (31–33) (Fig. 3A). This atlas was prepared from mice closest in age to those used in our study. Imaged brains were ~386 mm^3^ compared with ~500 mm^3^ of adult brains (34). Bright foci of viral RNA were detected in cell bodies using *deepBlink* (35). Background signal was manually excluded, and overdetection from overlapping planes was corrected. We enumerated infection foci and binned each according to all annotated regions available in the reference atlas.

**Fig 4:**
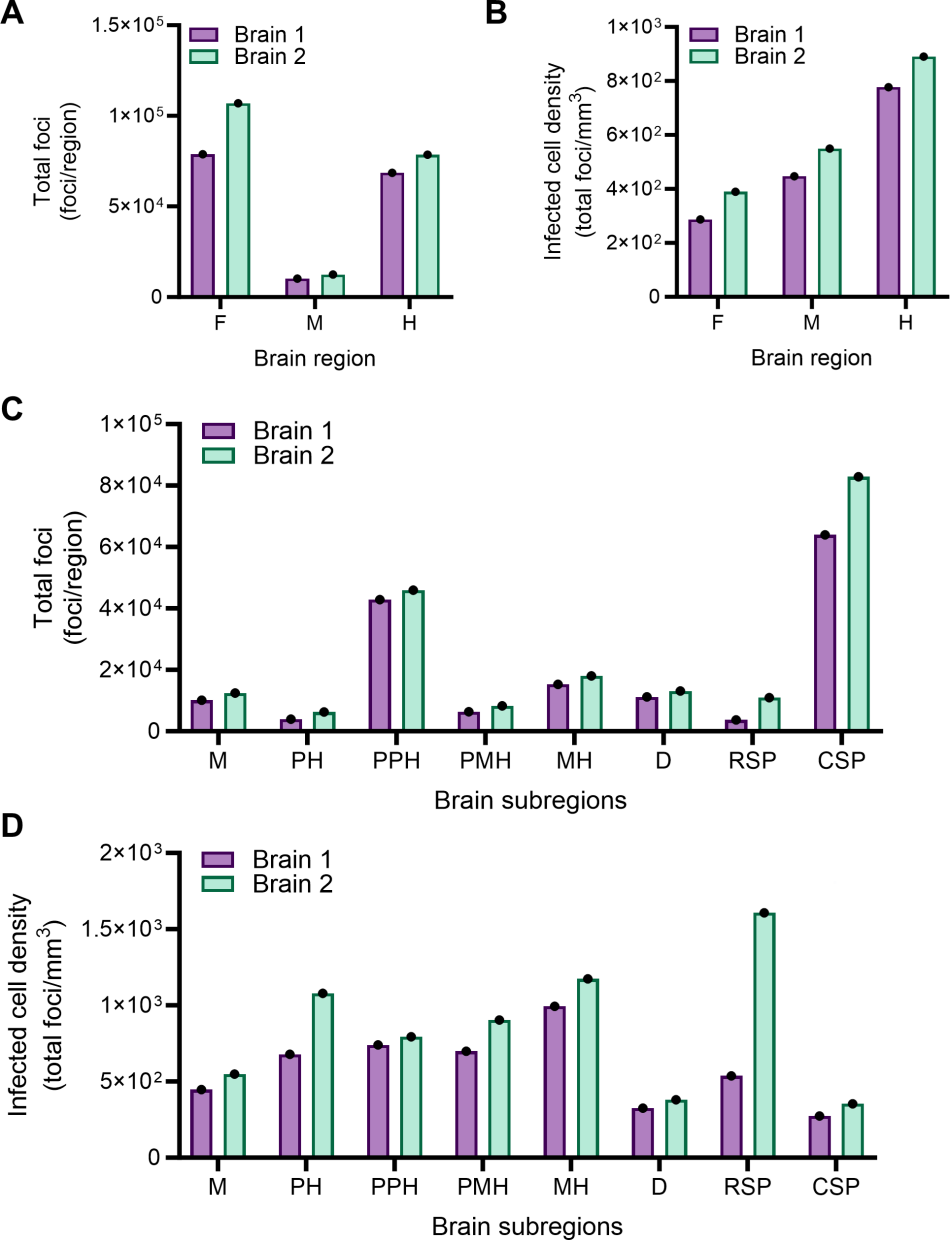
Alignment of two reovirus-infected 3D brain data sets to a P14 mouse brain atlas indicates that most regions of the brain are susceptible to infection. (**A–D**) Two-day-old WT mice were inoculated IC with phosphate-buffered saline (mock) or 1,000 PFU of reovirus strain T3SA+. Mice were euthanized at 7 days post-inoculation, and brains were resected and processed by MiPACT-HCR as in Fig. 2. Brain 1 was imaged using ribbon-scanning confocal microscopy (purple bars), and brain 2 was imaged using MesoSPIM (green bars). Mock-inoculated samples stained for viral RNA showed no background staining. Data were processed using Imaris software (Oxford Instruments). The 3D-reconstructed Allen Developing Mouse Brain Atlas–P14 (31–33) was aligned and overlaid with reovirus-infected brain data sets using the *brainreg* Python package. Virus-infected cell bodies within subregions of the 3D data set were enumerated using the *deepBlink* tool modified on U-Net architecture followed by the DBSCAN clustering method. Data are presented as total infection foci (**A and C**) for different anatomical regions or infectivity density (total foci count per region volume based on alignment) (**B and D**) for different brain regions. Each bar indicates data from a single brain. F, forebrain; M, midbrain; H, hindbrain; PH, pontine hindbrain; PPH, prepontine hindbrain; PMH, pontomedullary hindbrain; MH, medullary hindbrain; D, diencephalon; RSP, rostral secondary prosencephalon; and CSP, caudal secondary prosencephalon.

We first assessed infection counts by dividing the brain into three main regions: the forebrain, midbrain, and hindbrain, and further segmented these regions into subregions of interest (Fig. 3B). Distribution of infection foci in two brains imaged using different strategies was comparable, suggesting that distribution of HCR signal is nonrandom and dependent on viral tropism. The greatest number of infected cells was detected in the forebrain (Fig. 4A). However, when corrected for regional volume, the forebrain contained the lowest density of infection (Fig. 4B). Instead, reovirus infection appeared most dense in the hindbrain (Fig. 4B). To gain additional information about viral tropism, we quantified infection foci in the midbrain, pontine hindbrain, prepontine hindbrain, pontomedullary hindbrain, medullary hindbrain, diencephalon, rostral secondary prosencephalon, and caudal secondary prosencephalon subregions. In these experiments, the distribution of infection foci in the two brain samples correlated well for most regions tested (Fig. 4A through D). The rostral secondary prosencephalon (which includes the hypothalamus) subregion had the highest density of infection foci in brain sample 2 but also displayed the greatest variability between samples. Infection foci density was greatest on average in the medullary hindbrain and the pontine hindbrain (Fig. 4D), reflective of patterns in the hindbrain at large (Fig. 4B). Collectively, these results demonstrate that reovirus infection is detectable throughout the brain volume, and viral infection does not preferentially target a specific brain region or subregion.

### Reovirus glycan binding does not influence reovirus tropism in the CNS

**Fig 5:**
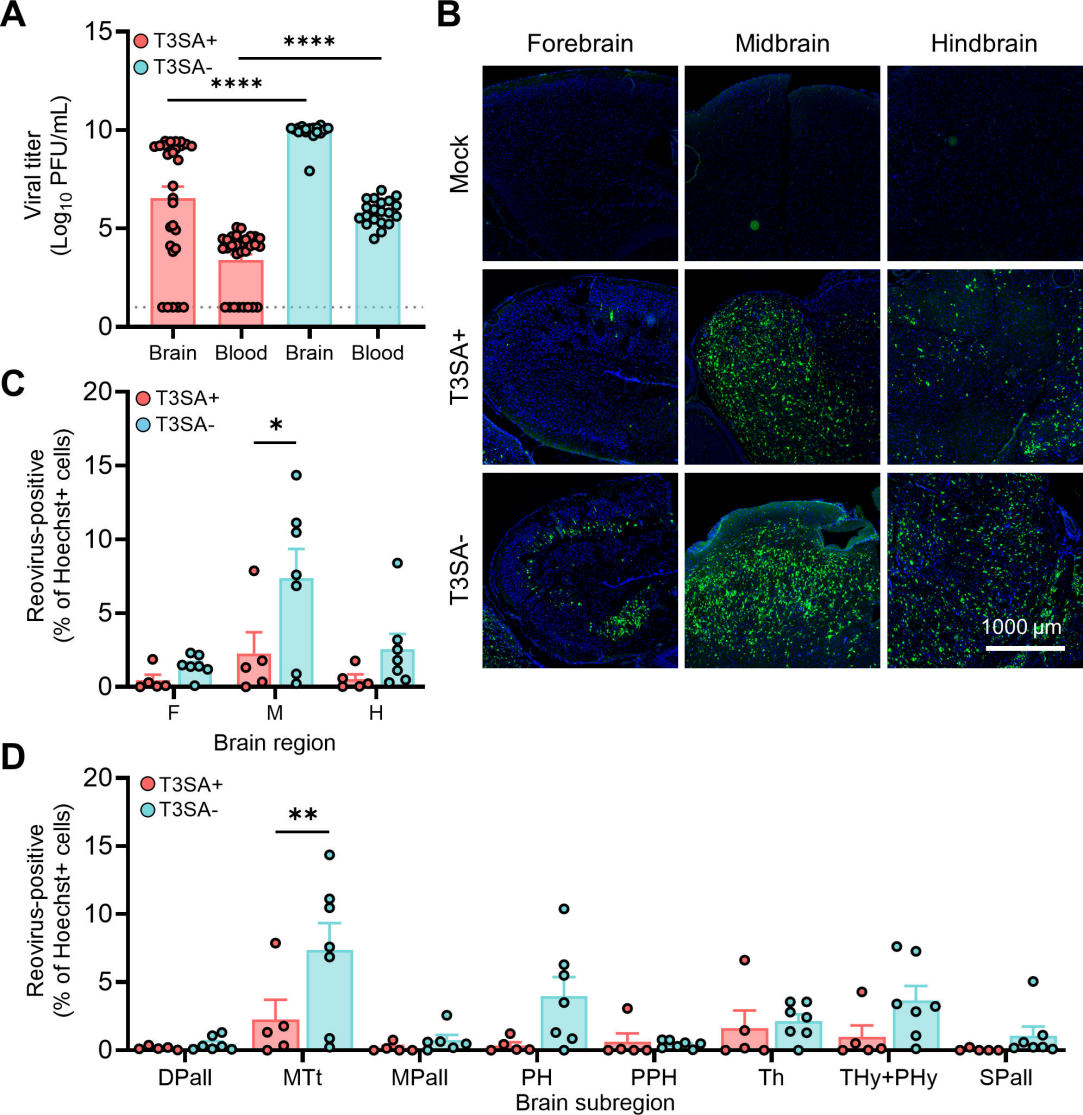
Impaired reovirus binding to sialic acid does not limit infection of sites in the brain. (**A–D**) Two-day-old WT mice were inoculated IC with phosphate-buffered saline (mock) or 1,000 PFU of reovirus strain T3SA+ or T3SA−. Mice were euthanized at 7 days post-inoculation, and brain tissue and whole blood were collected. (**A**) Titers of virus in homogenized left-brain hemispheres and blood were determined by plaque assay. Each symbol represents the viral titer from an individual animal. Brain*, N* = 29/29 (T3SA+/T3SA−); blood, *N* = 19/20 (T3SA+/T3SA−). Error bars indicate SEM. Values that differ significantly from T3SA+ by unpaired *t* test are indicated (****P < 0.0001). Dotted line indicates limit of detection. (**B–D**) Right-brain hemispheres (with contralateral hemisphere viral loads between 3.4e^8^ and 7.3e^9^) were fixed with formalin, embedded in paraffin, and sectioned sagittally. Tissue sections were probed for reovirus RNA by HCR, counterstained with Hoechst dye, and imaged using a Lionheart FX automated imager. (**B**) Representative images are shown for the indicated brain regions. Reovirus RNA is depicted in green; nuclei are depicted in blue. Scale bar, 1,000 µm. (**C and D**) Reovirus infection in established regions of interest. Infection foci (HCR-positive) from each region (**C**) or subregion (**D**) of mock-infected and reovirus-infected sections were enumerated using the Spot Detector tool within Icy software. Data are presented as the percentage of infected cells, wherein a reovirus-positive cell was determined by signal intensity greater than background defined using a mock-infected brain. *N* = 5/7 (T3SA+/T3SA−). Error bars indicate SEM. Values that differ significantly from T3SA+ by Sidak’s multiple comparisons test are indicated (*P < 0.05 and **P < 0.005). DPall, dorsal pallium; MTt, collicular midbrain tectum; MPall, medial pallium; PH, pontine hindbrain; PPH, prepontine hindbrain; Th, thalamus; Thy+PHy, hypothalamus; and SPall, subpallium.

Although viral receptors often dictate tropism in the infected host, previous studies have not identified attachment factors or receptors that contribute to patterns of reovirus infection in the CNS, perhaps because of the detection strategy used and the absence of quantitative histological analyses. We thought it possible that use of known receptors contributes to reovirus tropism in the brain. To determine whether attachment factor SA influences reovirus replication in the CNS, we compared viral infection in the brain using SA-binding strain T3SA+ and non-SA-binding (SA-blind) strain T3SA−. Two-day-old WT mice were inoculated with T3SA+ or T3SA− and euthanized 7 days post-inoculation. Brain tissue and blood were collected, and the brain was hemisected. The right-brain hemisphere was processed for IHC, and the left-brain hemisphere and blood were homogenized for viral load quantification (Fig. 5A). Average viral loads in the brain and blood were significantly higher in mice inoculated with T3SA− than those inoculated with T3SA+. These data reflect the detection of viral load at a single time point, late post-inoculation. However, these findings suggest that SA use is not required for reovirus replication in the brain or blood, consistent with previous studies (9, 14).

While whole-brain imaging established patterns of reovirus tropism, this technique is not amenable to high-throughput analyses. To understand the function of attachment factors more efficiently, we imaged reovirus infection using HCR in formalin-fixed and paraffin-embedded (FFPE) sections (FFPE-HCR) from right-brain hemispheres following inoculation of mice with T3SA+ or T3SA− (Fig. 5B through D). While precise viral load matching was not possible in these experiments, we compared samples from mice infected with T3SA+ or T3SA– that were within a 10-fold viral load range in the contralateral brain hemisphere. Sections near the brain midline and at comparable brain depth were selected by comparing hippocampi, an easily distinguishable structure in the brain. FFPE-HCR samples were processed using automated imaging and image stitching (Fig. 5B). We captured abundant infection foci in all three brain regions assessed in mice infected with either virus strain. In some cases, infection appeared to target axons. For example, in the forebrain of mice infected with T3SA−, thin structures that stained for viral RNA extended beyond the globular cell bodies (Fig. 5B). These findings suggest that SA engagement does not contribute to reovirus tropism.

To quantify reovirus infection, we developed a strategy to enumerate infection foci in subregions of the brain. We designed eight regions of interest (ROIs) by mapping a plane of the Allen Developing Mouse Brain Reference Atlas (P14) (33, 36) onto a brain sample from a mock-infected mouse and outlined portions of major subregions of the brain (Fig. S1A and B). For each FFPE-HCR sample, identical ROIs were manually placed using the hippocampus as a landmark. Icy software was used to enumerate the total number of HCR-positive cells in each ROI relative to mock-infected controls. Reovirus T3SA+ and T3SA− infected all three major brain regions (Fig. 5C). This finding is consistent with results from the 3D data sets (Fig. 4) and previous findings (14). When we evaluated infection in brain subregions, both strains produced the highest percent infection in the collicular midbrain tectum compared with the other brain subsections assessed (Fig. 5D). T3SA− infected the midbrain more efficiently than T3SA+ and trended toward more efficient infection in the forebrain and hindbrain. This difference may be attributable to the higher average viral loads in the contralateral brain hemispheres of mice inoculated with SA-blind virus T3SA−. We did not identify a subregion of the brain in which infection was dependent on SA binding. Together, these results suggest that glycan binding is not required for reovirus neurotropism but instead may modestly attenuate infection.

### PirB does not influence reovirus tropism in the CNS

**Fig 6:**
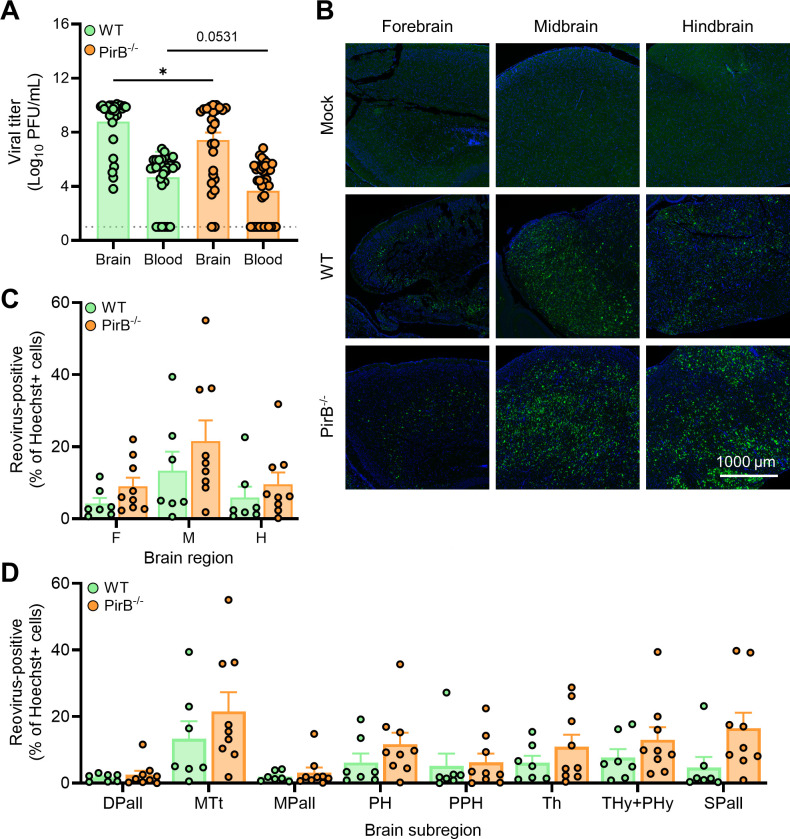
PirB expression does not dictate reovirus infection patterns in the brain. (**A–D**) Two-to-three-day-old WT or PirB^-/-^ mice were inoculated IC with phosphate-buffered saline (mock) or 200 PFU of reovirus strain T3SA−. Mice were euthanized at 7–8 days post-inoculation, and brain tissue and whole blood were collected. (**A**) Titers of the virus in the homogenized left-brain hemisphere and blood were determined by plaque assay. Each symbol represents the viral titer from an individual animal. Brain, *N* = 28/27 (WT/PirB^-/-^); blood, *N* = 31/35 (WT/PirB^-/-^). Error bars indicate SEM. Values that differ significantly from WT by unpaired *t* test are indicated (*P < 0.05). Dotted line indicates limit of detection. (**B–D**) Right-brain hemispheres (with contralateral hemisphere viral load between 4.17e^8^ and 1.4e^10^) were fixed in formalin, embedded in paraffin, and sectioned sagittally. Tissue sections were probed for reovirus RNA by HCR, counterstained with Hoechst dye, and imaged using a Lionheart FX automated imager. (**C**) Representative images are shown. Reovirus RNA is depicted in green; nuclei are depicted in blue. Scale bar, 1,000 µm. (**C and D**) Reovirus infection in established ROIs. Infection foci (HCR-positive) from each region (**C**) or subregion (**D**) of mock-infected and reovirus-infected mouse sections were enumerated using the Spot Detector tool within Icy software. Data are presented as the percentage of infected cells, wherein a reovirus-positive cell was determined by signal intensity greater than background defined using a mock-infected brain. *N* = 7/9 (WT/PirB^-/-^). Error bars indicate SEM. Values that differ significantly from WT by Sidak’s multiple comparisons test are indicated. DPall, dorsal pallium; MTt, collicular midbrain tectum; MPall, medial pallium; PH, pontine hindbrain; PPH, prepontine hindbrain; Th, thalamus; Thy+PHy, hypothalamus; and SPall, subpallium.

PirB is a reovirus receptor (13) expressed on neurons in regions that correlate with reovirus tropism in the brain (23). Following intracranial inoculation of WT control mice (NspPirB^+/+^) or neural-specific PirB-null mice (NspPirB^-/-^) with reovirus T3SA− at a low dose (20 PFU), survival of PirB-null mice was enhanced, but there were no gross differences in viral tropism in the two mouse strains (13). To determine whether there are subtle differences in reovirus neurotropism dependent on PirB expression, we inoculated WT and PirB^-/-^ mice with 200 PFU of T3SA−. This strain of virus enables us to remove any confounding interactions with SA. At 7–8 days post-inoculation, mice were euthanized, and brain tissue and blood were collected. Viral loads of left-brain hemispheres and blood (Fig. 6A) were determined by plaque assay. Right-brain hemispheres were fixed and processed for FFPE-HCR. There was significantly more viral load in the brain, and viral load trended higher in the blood of WT mice compared to PirB^-/-^ mice inoculated with reovirus. These results are consistent with published findings (13).

To test whether PirB expression influences reovirus tropism in the brain, we used FFPE-HCR to quantify reovirus infection in brain sections from inoculated WT and PirB-null mice within a range of 100-fold viral load in the contralateral brain hemisphere. Reovirus infection foci were observed in all three major regions of the brain and had similar staining patterns in WT and PirB^-/-^ mice. These trends included viral RNA staining of neuronal cell bodies and axons (Fig. 6B). There was no significant difference in detection of infection foci in either WT or PirB^-/-^ mice at either the regional (Fig. 6C) or subregional level (Fig. 6D). In both conditions, infection foci were detected at the highest percentage in the midbrain region, specifically the collicular midbrain tectum (Fig. 6C and D). Therefore, for both SA (Fig. 5) and PirB (Fig. 6) binding, the midbrain appears to be a major site for reovirus infection in the CNS when infection is determined using 2D imaging modalities. When accounting for regional volume using 3D techniques, the infection density in the hindbrain surpasses that of the midbrain region. Together, these data indicate that reovirus neurotropism is not dependent on binding to either SA or PirB.

## DISCUSSION

The genetic linkage of reovirus attachment protein σ1 to neurotropism (7, 9) suggests that receptors or other entry mediators determine serotype-specific differences in reovirus infection of neurons. However, a comprehensive understanding of reovirus tropism or how reovirus attachment factors and entry receptors, such as sialylated glycans and PirB, contribute to reovirus neurotropism is not available. In this study, we used whole-brain imaging MiPACT-HCR-SWITCH and identified reovirus infection throughout the forebrain, midbrain, and hindbrain. Using FFPE-HCR, we found that engagement of SA or PirB is not required for reovirus infection in the brain. Collectively, these data support the conclusion that reovirus infection of neurons is determined by attachment factors or entry receptors that have thus far not been identified.

While reovirus infects diverse regions in the murine brain (6, 7, 9, 11, 12), previous studies have identified brain subregions susceptible to reovirus using 2D IHC, which captures infection within ~0.1% of the total brain volume. As such, this strategy does not consider the volume of the tissue and thus may not identify major regions infected by reovirus throughout the brain. In addition, previous studies used reovirus antiserum staining (6, 7, 9, 11, 12), which could detect reovirus debris phagocytosed by CNS-resident immune cells and thus not indicate active infection. To expand an understanding of neurotropism to the entire brain volume, we developed an HCR-based approach that detects late stages of infection (Fig. 1) to characterize reovirus neurotropism in 2D and 3D by staining for viral RNA.

To identify sites of reovirus neurotropism in three dimensions, we established MiPACT-HCR-SWITCH to optically clear tissue and stain viral RNA within an intact brain hemisphere (Fig. 2A and C through E). MiPACT effectively preserves tissue architecture (27), including structures like axons that can extend across the brain volume (Fig. 2E and F). Using this approach, we were able to resolve viral RNA staining extending along thin, axon-like structures, suggesting that reovirus uses these elements to replicate or disseminate (Fig. 2D through F). We found that viral RNA staining was most intense at the periphery of the brain tissue (Fig. 2C). Perhaps the inoculation strategy used here contributes to this staining pattern since the tissue exterior is occupied by blood vessels that could be used for viral dissemination within the brain and may facilitate greater infection at the periphery. Alternatively, HCR reactions may be less efficient in the deepest regions of the brain. However, the MiPACT-HCR-SWITCH strategy effectively stains viral RNA within a majority of the murine brain hemisphere and enables single-cell resolution of reovirus RNA in substantial detail.

While visualizing reovirus RNA throughout the brain volume provided valuable insights, it also enabled us to establish a new workflow to quantify susceptible brain regions and thus define major targets of infection, some of which have been underreported. We annotated 3D data sets (Fig. 3A) to quantify viral tropism in major regions and subregions (Fig. 3B). Many of our findings are concordant with previous IHC-based studies of reovirus infection in the murine CNS (6, 7, 9, 11, 12) (Fig. 4). However, we observed that the midbrain was heavily infected (Fig. 4B), which had not been a previously recognized feature of reovirus neurotropism. Prominent viral infection in the midbrain could be explained by intracranial inoculation, which may introduce the virus inoculum directly into this region and allow the virus to simultaneously infect multiple adjacent cells. Alternatively, neurons in the midbrain are heavily interconnected with other brain regions targeted by reovirus, such as cortical layers IV and V and the thalamus (37), and thus, virus may be shuttled to the midbrain from other susceptible areas in the CNS. Therefore, the unbiased imaging approach used here allowed us to identify underreported areas of infection and form new hypotheses about viral dissemination in the brain, which could be probed in future studies using stereotaxic inoculations.

We found that the most concentrated region of reovirus infection is in the hindbrain (Fig. 4B), and more specifically, the medullary and pontine hindbrain (Fig. 4D). During murine development, the pontine hindbrain communicates with other sensory neurons and interacts with regions of the medullary hindbrain (38). Some neurons in the medullary and pontine hindbrain directly interact through synaptic connections (38), suggesting that reovirus could be transported directly between these neural structures. Thus, our 3D data reveal that reovirus targets several regions of the brain, with the most prominent infection in the midbrain and medullary and pontine hindbrain.

Using MiPACT-HCR-SWITCH, we identified major sites of reovirus infection in mice. 3D microscopy of this scale requires deep volumetric imaging, programming knowledge, and large (4 terabytes or more) data storage. This strategy can be used to define areas of interest that can then be probed further using a higher-throughput approach such as 2D imaging. As volumetric microscopy and bioimaging quantification become more accessible, these strategies may be more amenable to quickly testing hypotheses about neurotropic viral infections, among other topics.

After identifying regions targeted by reovirus in the brain of WT mice, we sought to complement our 3D approach by varying virus strain and host receptor expression and staining for viral RNA in 2D brain slices. These studies enabled us to test how reovirus receptors influence patterns of neurotropism. Reovirus engages SA as an initial adhesion-strengthening step prior to receptor-mediated endocytosis to initiate infection (18). SAs are broadly expressed throughout the nervous system (39) in expression patterns that mimic reovirus neurotropism. We found that a non-SA-binding reovirus strain, T3SA−, produces higher viral loads in the brain and blood relative to its SA-binding counterpart, T3SA+, at the dose and time point tested in this study (Fig. 5A). There also was greater variability in viral loads in animals inoculated with T3SA+ relative to those inoculated with T3SA−, which may reflect biological variability in mouse experiments or perhaps a virus-driven host response. Interactions between reovirus and SA potentiate apoptosis (40) and may contribute to viral clearance and thus lower viral loads in mice infected with T3SA+. In addition, activation of apoptosis by T3SA+ may reduce the number of cells containing viral RNA that can be identified. It is also possible that engagement of SA impedes reovirus entry into neurons, perhaps by increasing adherence to non-susceptible cells or resident immune cells. Using FFPE-HCR, we found that T3SA− infected the midbrain more efficiently than T3SA+ (Fig. 5C and D). Infection of other regions, such as the pontine hindbrain and the hypothalamus, trended toward similar results, suggesting that the influence of SA on neurotropism may vary by brain region. However, we were unable to identify any region that differed strikingly in susceptibility to T3SA− and T3SA+. Thus, reovirus neurotropism does not appear to be dependent on the capacity to bind SA.

Reovirus engages entry receptors such as PirB to facilitate endocytosis (13). PirB is expressed throughout the CNS in a pattern that overlaps with reovirus neurotropism (23). PirB expression is required for maximal titers of reovirus in the murine brain (13), but it was not known whether PirB contributes to reovirus neurotropism. In our study, viral loads in the brain of WT mice were significantly greater than those in PirB-null mice 7 days post-inoculation (Fig. 6A). These results are concordant with previous studies investigating the function of PirB in reovirus pathogenesis (13). Staining for reovirus in histological sections revealed no difference in viral infection in all brain regions assessed (Fig. 6B through D). These results suggest that while PirB functions in the efficiency of viral uptake and virulence in mice, PirB is not a major tropism determinant. Like reovirus, Venezuelan equine encephalitis virus infects regions of the brain, including the midbrain, and similarly, infection in some regions does not require viral receptor LDLRAD3 (41). This finding raises the possibility that neurons in specific brain regions are susceptible to neurotropic virus infection independent of the expression of viral receptors, at least those that are known.

Our studies contribute to an understanding of how reovirus targets the murine brain to cause encephalitis. To better understand the host factors that influence reovirus neurotropism, future studies are required to define receptors or other host factors unique to susceptible cells. It is possible that cell-intrinsic elements, such as metabolism or physical connections between cells like synapses, dictate reovirus neurotropism. Studies using MiPACT-HCR-SWITCH to amplify nucleic acid in multiplexed experimental applications can be used to define unique populations of cells infected by the virus or identify viral and host transcripts in infected cells. In addition, whole organ imaging strategies can be applied to other neurotropic viruses to identify shared susceptible cell types or to test infection of neural circuits using neuronal tracking applications (42). An understanding of how and where viruses spread in the brain will enhance knowledge of vulnerabilities of this tissue and potentially identify new therapeutic targets.

## MATERIALS AND METHODS

### Cells and viruses

Spinner-adapted L929 fibroblasts (originally obtained from Dr. Bernard Fields) were maintained in suspension in Joklik’s minimal essential medium supplemented to contain 5% FBS, 2 mM L-glutamine, 100 U/mL penicillin and streptomycin, and 250  ng/mL amphotericin B. HeLa S3 cells (43) were maintained in Dulbecco’s modified eagle’s medium supplemented to contain 10% FBS. Reovirus strains T3SA+ and T3SA− were recovered using plasmid-based reverse genetics (44). Reovirus purification and viral load quantification were conducted as described previously (45, 46).

### Hybridization chain reaction probe design

Ten 52-base-pair regions were selected as virus-specific HCR probe pairs (referred to as “probes”) in the open-reading frame of the reovirus T1 Lang (T1L) S3 gene segment, which is present in both the T3SA+ and T3SA− recombinant strains (26). Complementary sequences of the first 25 base pairs of a region were appended with HCR 3.0 B1 amplifier sequences (“hairpins,” Molecular Instruments, Inc.), which include antiparallel sequences of the last 25 base pairs of that region (Table 1). Probes were synthesized by Integrated DNA Technologies and purified by desalting.

### Immunofluorescence and HCR staining

HeLa cells were seeded onto poly-D-lysine-coated glass coverslips, incubated overnight, and adsorbed with reovirus at an MOI of 100 PFU/cell diluted in medium at room temperature (RT) for 1 h. The inoculum was replaced with fresh medium, and at various intervals post-adsorption, supernatants were removed, and cells were fixed with 4% paraformaldehyde in phosphate-buffered saline (PBS) at RT for 30 min. Cells were washed with PBS and processed for HCR following the RNA-FISH “Mammalian cells on a slide” protocol provided by Molecular Instruments, Inc. Cells were permeabilized with 0.1% Triton-X at RT for 1 h and washed with PBS. Samples were incubated with probe-hybridization buffer (Molecular Instruments, Inc.) at 37°C for 30 min and subsequently incubated with 4 nM probes in probe-hybridization buffer at 37°C overnight. Samples were washed with probe-wash buffer (Molecular Instruments, Inc.) four times at 37°C for 15 min each and with 5× SSC-T [saline-sodium citrate (SSC) buffer (Invitrogen 15557036) with 10% Tween-20] three times at RT for 5 min. HCR hairpins (Molecular Instruments, Inc.) were incubated at 95°C for 90 s and cooled at RT for 30 min in the dark. Cells were incubated with probe-amplification buffer (Molecular Instruments, Inc.) at RT for 30 min and subsequently incubated with 60 nM cooled hairpins in probe-amplification buffer (Molecular Instruments, Inc.) at RT overnight. Samples were washed with 5× SSC-T for 2 × 5 min, 2 × 30 min, and 1 × 5 min. Cells were fixed with 4% paraformaldehyde at RT for 20 min. Cells were blocked with 5% bovine serum albumin (BSA) in PBS at RT for 30 min and consecutively stained with rabbit reovirus-specific antiserum (9) and anti-rabbit Alexa Fluor-labeled secondary antibody (Invitrogen) diluted in PBS-BGT (0.5% BSA, 0.1% glycine, and 1% Tween-20 containing PBS) at RT for 1 h. Nuclei were counterstained with Hoechst 34580 dye. Following incubation with antibody or Hoechst stain, samples were washed with PBS three times for 5 min each. Glass coverslips were adhered to glass slides using Aqua Poly/Mount (Polysciences, Inc.). Cells were imaged using a Leica SP8 confocal microscope equipped with a 63× oil lens objective and processed using Icy software (47).

### Animal studies

All mice used in this study were maintained in a specific pathogen-free vivarium at the University of Pittsburgh. Experiments using mice included approximately equivalent numbers of males and females. Wild-type C57BL/6J or PirB^-/-^ mice of the same background were genotyped as described (13).

Two-to-three-day-old mice were inoculated IC in the right cerebral hemisphere with 5 µL containing 1,000 or 200 PFU of reovirus diluted in PBS using a 30-gauge needle with a Hamilton syringe. Viral titer in the inoculum was quantified by plaque assay and experiments using an inoculum viral concentration ±3-fold were accepted. Mice were euthanized at 7–8 days post-inoculation, and brains and whole blood were collected. Brains were hemisected along the longitudinal fissure. When viral titers in both hemispheres were determined, the right or left hemispheres were frozen in 1 mL of PBS. For the analysis of both viral load and tropism, left brain hemispheres were frozen in 1 mL of PBS for the determination of viral titers and right (inoculated) brain hemispheres were fixed in 10% neutral-buffered formalin for a minimum of 48 h. Brain and blood samples were frozen, thawed, and homogenized using a TissueLyser (Qiagen) twice prior to viral titer determination by plaque assay.

### Passive brain tissue clearing using MiPACT

MiPACT-HCR approaches used here were modified from DePas et al., (27). For clearing, each right-brain hemisphere was first fixed in 40 mL of 10% neutral-buffered formalin at 4°C for 72 h. Fixative was refreshed at 36 h. Fixed tissue was washed twice with PBS on a platform shaker rotating at 80 rpm at RT for 2 h. Brain tissue was subsequently incubated at 4°C overnight in embedding mixture [44% (vol/vol) of 29:1 acrylamide:bis-acrylamide (BioRad, #1610146) and 0.25% (wt/vol) VA-044 hardener (FUJI Film, #223-02112) in PBS] with sufficient volume to cover the tissue. The embedding mixture was replaced, and samples were transferred to glass test tubes with rubber stoppers. Using a vacuum, headspace oxygen was removed from tubes, and samples were gently sonicated using a Branson 2800 Ultrasonic water bath to remove air bubbles. Samples were incubated at 37°C for 3–4 h to polymerize acrylamide. Excess polymer was trimmed using a razor blade. Samples were incubated in 0.1 M methylimidazole (pH 8.4, Sigma-Aldrich, #M50834) at RT for 30 min and subsequently incubated in 0.1 M methylimidazole supplemented to contain 0.1 M EDC (Thermo, #PG82073) and 0.09 M 5-ethylthio-1H-tetrazole (Sigma-Aldrich, #493805) at 37°C for 1 h. Samples were placed in tissue macro-cassettes (Fisherbrand, #15-182-706) and suspended on a steel wire in a beaker containing 1 L of 8% SDS-containing PBS (pH 6.8) on a stir plate at 37°C for 14 days. SDS buffer was replaced every 3–5 days. Cassettes were rinsed with de-ionized water for 2–3 min and samples were washed with 40 mL of PBS at RT for 3 h or until SDS was visually removed.

### Staining cleared brain tissue using SWITCH-HCR

Cleared tissue was incubated in ab-OFF buffer (5 mM SDS in PBS) at 37°C for 3 h. Buffer was replaced, supplemented to contain 0.1 µM probes, and incubated at 37°C for 4 days with gentle agitation. Hybridization reactions were activated by replacing ab-OFF buffer with probe-hybridization buffer (Molecular Instruments, Inc.) containing 0.1 µM probes, and brains were incubated at 37°C for 2 days with gentle agitation. Samples were washed with prewarmed probe-wash buffer (Molecular Instruments, Inc.) at 37°C for 3 h. Samples were light-protected and incubated with ab-OFF buffer supplemented to contain 0.13 µM B1 hairpins at RT for 4–7 days with gentle agitation and then incubated with probe-amplification buffer (Molecular Instruments, Inc.) at RT for 2 days with gentle agitation. Samples were washed with 337.5 mM NaCl at 37°C for 1 day. Samples were incubated in CUBIC R2 solution (75 mL water, 125 g urea, 250 g sucrose, and 380 µL Triton X-100) at RT for 2–4 days and refreshed with CUBIC R2 prior to imaging.

### Whole-brain imaging

A custom MesoSPIM microscope fitted with a Photometrix Kinetix camera and a Nikon AZ100 2× objective was used to image stained brain hemispheres (30). This MesoSPIM uses two light sheets, from the left and the right, to illuminate both sides of the sample. Brain tissue was mounted in a quartz cuvette in CUBIC R2 and submerged in a larger cuvette containing mounting solution with a refractive index of 1.467 (TCI Chemicals, #M3292). Images were acquired with a final magnification of 16× and voxel resolution of 0.5 × 0.5 × 5 µm (X, Y, Z). Fluorescence was captured in the 561 nm channel with a 595/44 RFP filter in a total of 77 Z-stacks, each containing 1,872 images. Z-stacks were saved as BigTIFF files, converted to Imaris (Oxford Instruments) files using ImarisFileConverter (Bitplane, version 10.0.0), and stitched using ImarisStitcher (Bitplane, version 10.0.0). 3D reconstructions of the samples were rendered using Imaris software (Bitplane, version 10.0.0).

The Caliber I.D. (Andover, MA, USA) RS-G4 microscope (“Ribbon-scanning confocal”) fitted with a Nikon CF190 20× glycerol-immersion objective was also used to image stained brains (48). Fluorescence was captured in the 488 nm channel with a Semrock FF01-450/70 filter and in the 561 nm channel with a Semrock FF01-630/69 filter. The 561 nm laser intensity increased linearly throughout the tissue, while the 488 nm laser was kept constant. The sample was imaged with a voxel resolution of 0.492 × 0.492 × 6.86 µm (X, Y, Z) and a total of 754 Z-slices. Ribbons were saved as TIFF files, assembled using a computational cluster as described (29), and converted to the Imaris (Oxford Instruments) format.

### Quantitative 3D brain image analysis

Image analysis was conducted for 4 × 4 × 12 µm/pixel (X, Y, Z) representative images (Imaris, Oxford Instruments). Fully automated methods for 3D spot detection and alignment to reference atlas were used (31). Cell bodies were detected using a deep-learning spot detection method *deepBlink* with a pretrained particle model (35, 49). Overdetection in adjacent Z-planes was corrected using the DBSCAN (50) clustering method provided by Python’s scikit-learn library (51) to cluster multiple detections and remove all but the centroid. Detected foci were mapped to brain regions by aligning the brain to a community-contributed 3D-reconstructed Allen Developing Mouse Brain Atlas (P14) (31–33) available through the BrainGlobe atlas API (52) using *brainreg* software (53). Brain region nomenclature used throughout the study is derived from P14 atlas. The autofluorescence signal collected in the ribbon-scanned data set was used for alignment, whereas the MesoSPIM data set only contained a viral signal. Bright signals that distorted alignment to the atlas were overcome by training the Noise2Noise-based neural network (54) on signal-autofluorescence images from other experiments to reduce signal appearance in the image. Every infection focus corresponding to a brain structure was saved into a single CSV file per brain. Further aggregation of the number of spots in specific brain regions was accomplished using the Pandas Python library (55).

### Staining of histological sections for viral RNA using HCR

Brain hemispheres used for histology were fixed and embedded in paraffin, and sections (5 µm) were adhered to glass slides. Brain sections from approximately the same region across samples were matched according to hippocampus structure. Sections were incubated at 60°C for 1 h, serially deparaffinized, and incubated with antigen-retrieval buffer [10 mM sodium citrate buffer (pH 6.0)] at 95°C for 15 min. HCR assays were conducted following the manufacturer’s instructions (“FFPE tissue sections,” Molecular Instruments, Inc.). Samples were incubated with probe-hybridization buffer (Molecular Instruments, Inc.) at 37°C for 30 min and subsequently incubated with 4 nM probes diluted in hybridization buffer at 37°C overnight. Slides were washed sequentially with probe-wash buffer (Molecular Instruments, Inc.) in 5X SSC-T (75:25, 50:50, 25:75, and 0:100 of wash buffer:5X SSC-T) at 37°C for 15 min each. Samples were incubated with probe-amplification buffer (Molecular Instruments, Inc.) for 30 min at RT and then incubated with amplification buffer and 60 nM snapcooled hairpins at RT overnight. Nuclei were counterstained using Hoechst dye in PBS. Samples were rinsed three times with PBS for 5 min each. Slides were overlaid with glass coverslips using Aqua-Poly/Mount (PolySciences, Inc.). Stained slides were scanned using a Lionheart FX Imager. Analysis was conducted using Icy Software (version 2.4.2.0) (47). Images were rotated to obtain identical orientations. ROIs were established by overlaying and aligning an image of a Hoechst-stained mock-infected sagittal brain section according to hippocampal architecture with a partially translucent image from a similar brain depth using the Allen Developing Mouse Brain Reference Atlas (P14) (Fig. S1A and B) (33, 36). Brain ROIs were outlined using the ROI function. ROIs were saved to preserve size, and identical ROIs were used for all samples. ROIs were placed on images of tissue and aligned according to tissue landmarks for each ROI, such as hippocampus, isocortex layers, or cell morphology. The dorsal pallium and midbrain were represented by two ROIs each, and the data sum for both was determined. The Icy Spot Detection function (56) (bright spots detector; 3 pixels at 150% sensitivity; size filtering, 25–150 μm) was used to enumerate total foci using Hoechst+ and viral RNA+ sites. PBS-inoculated controls were used to establish zero infectivity. Infectivity was quantified as a percentage of total cell foci: percent-infected cells = (viral RNA+ spots) ÷ (Hoechst+ spots) * 100.

### Determination of histological slice volume

The percentage of total brain volume represented by a histological slice (5 µm) was determined using the total Allen Developing Mouse Brain Reference Atlas (P14) length of approximately 5,125 µm (33, 36).

### Statistical analysis

All enumerated data were analyzed using GraphPad Prism version 9.5.1. The number of experimental repeats and statistical tests applied for each assay are provided in the figure legends. Differences in pairwise comparisons were considered statistically significant when *P* values were less than 0.05.

## SUPPLEMENTAL MATERIAL

Supplemental material msphere.00629-24-s0001.pdf Figure S1 and legends for Movies S1A and S1B.

Movie S1A msphere.00629-24-s0002.mp4 Imaged volume of reovirus-infected brain tissue visualized using MiPACT-HCR.

Movie S1B msphere.00629-24-s0003.mp4 Imaged volume of reovirus-infected brain tissue visualized using MiPACT-HCR.

ASM does not own the copyrights to Supplemental Material that may be linked to, or accessed through, an article. The authors have granted ASM a non-exclusive, world-wide license to publish the Supplemental Material files. Please contact the corresponding author directly for reuse.
