# Supplementary material for: Sialic acid and PirB are not required for targeting of neural circuits by neurotropic mammalian orthoreovirus
Source: mSphere. 2024 Sep 25;9(10):e00629-24. doi: 10.1128/msphere.00629-24 (PMC11540169; doi:10.1128/msphere.00629-24)
Supplement: Supplemental material — Figure S1 and legends for Movies S1A and S1B. [file msphere.00629-24-s0001.pdf]

Sialic acid and PirB are not required for viral targeting of neural circuits by neurotropic mammalian orthoreovirus

Kira A. Griswold,[1,2] Iaroslavna Vasylieva,[3] Megan C. Smith, [3] Kay L. Fiske, [2,4] Olivia L. Welsh,[2,4] Alexa N. Roth,[2,4] Alan M. Watson, [3] Simon C. Watkins,[3] Danica M. Sutherland,[2,4,#] Terence S. Dermody[1,2,4,#]

[1] Department of Microbiology and Molecular Genetics, University of Pittsburgh School of Medicine, Pittsburgh, Pennsylvania, USA

[2] Institute of Infection, Inflammation, and Immunity, UPMC Children's Hospital of Pittsburgh, Pittsburgh, Pennsylvania, USA

[3] Department of Cell Biology and Center for Biologic Imaging, University of Pittsburgh, Pittsburgh, Pennsylvania, USA

[4] Department of Pediatrics, University of Pittsburgh School of Medicine, Pittsburgh, Pennsylvania, USA

Running Head: Reovirus neurotropism in the central nervous system

# Address correspondence to: Danica M. Sutherland, danica.sutherland@pitt.edu, and Terence S. Dermody, terence.dermody@chp.edu

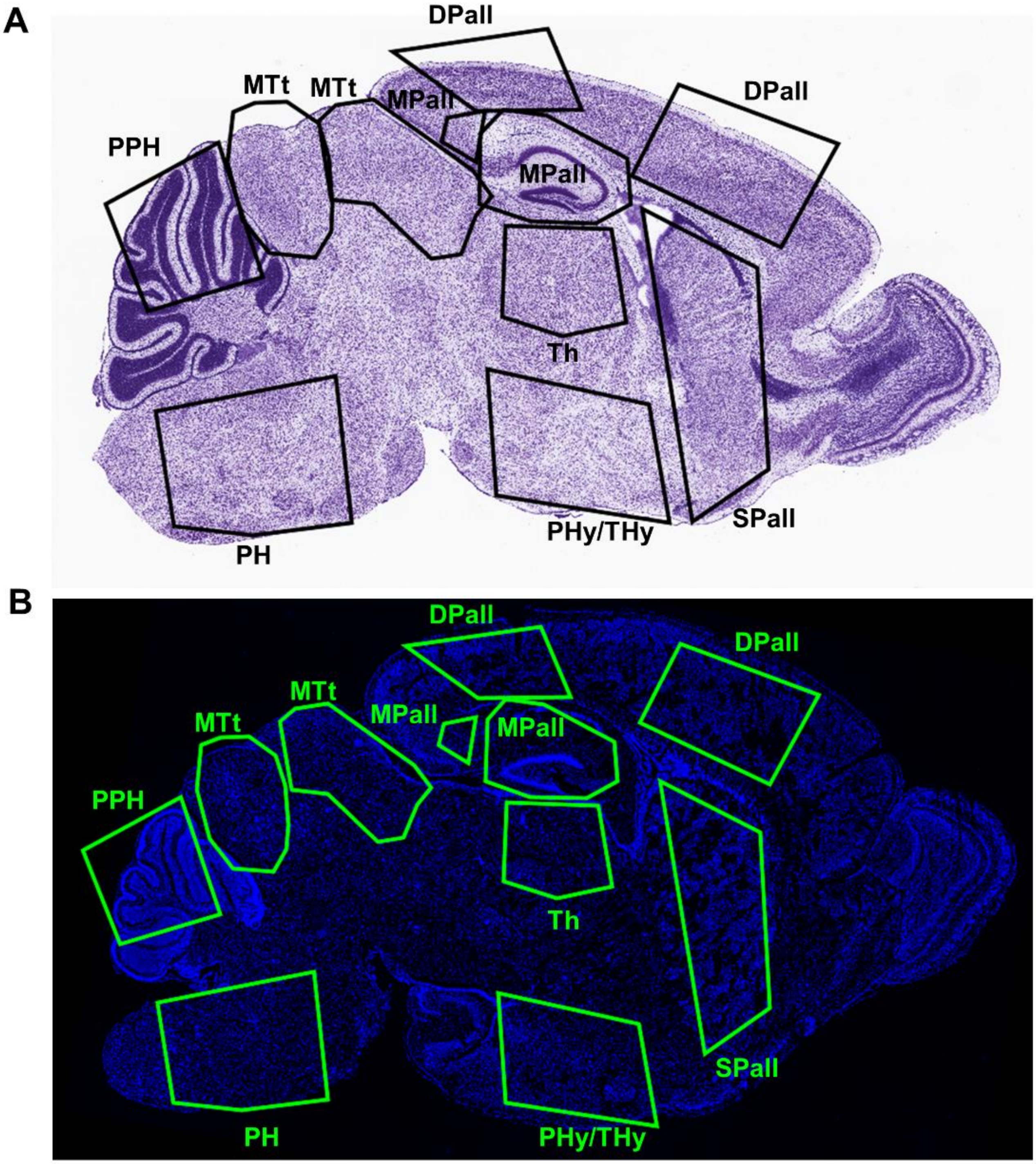

FIG S1 Histological mapping approach using a mouse brain atlas to quantify regional relative infection density. (**A**) Nissl-stained sagittal brain slice from the Allen Developing Mouse Brain Reference Atlas (Allen Developing Mouse Brain Reference

Atlas P14, developingmouse.brain-map.org and atlas.brain-map.org/) overlaid with regions of interest (ROI). Identical ROIs were individually overlaid onto all imaged sections according to brain landmarks (1,2). (**B**) ROIs (green outlines) overlaid on a Hoechst-dye-stained brain slice from a mock-infected animal. Brain regions are indicated. DPall, dorsal pallium; MTt, collicular midbrain tectum; MPall, medial pallium; PH, pontine hindbrain; PPH, prepontine hindbrain; Th, thalamus; Thy+PHy, hypothalamus; SPall, subpallium. Some regions (such as DPall, MPall, and MTt) are quantified from multiple ROIs.

Supplemental Movie S1A. Imaged volume of reovirus-infected brain tissue visualized using MiPACT-HCR. Two-day-old WT mice were inoculated IC with 1000 PFU T3SA+, and right-brain hemispheres were processed for MiPACT-HCR as described in FIG 2. Tissue was imaged using MesoSPIM microscopy and processed into a video using Imaris software (Oxford Instruments). Reovirus RNA is depicted in green. Movie of sagittal optical sections across entire brain hemisphere. Scale bar, 1000 μm.

Supplemental Movie S1B. Imaged volume of reovirus-infected brain tissue visualized using MiPACT-HCR. Two-day-old WT mice were inoculated IC with 1000 PFU T3SA+, and right-brain hemispheres were processed for MiPACT-HCR as described in FIG 2. Tissue was imaged using MesoSPIM microscopy and processed into a video using Imaris software (Oxford Instruments). Reovirus RNA is depicted in green. Max projection of z-stacks formatted for movie demonstrating reovirus RNA staining at cell resolution in 3D. Scale bar, 50 µm.

REFERENCES

1. Allen Institute for Brain Science (2013). Allen Developing Mouse Brain Reference Atlas. Available from developingmouse.brain-map.org
2. Thompson CL, Ng L, Menon V, Martinez S, Lee CK, Glattfelder K, Sunkin SM, Henry A, Lau C, Dang C, Garcia-Lopez R, Martinez-Ferre A, Pombero A, Rubenstein JLR, Wakeman WB, Hohmann J, Dee N, Sodt AJ, Young R, Smith K, Nguyen TN, Kidney J, Kuan L, Jeromin A, Kaykas A, Miller J, Page D, Orta G, Bernard A, Riley Z, Smith S, Wohnoutka P, Hawrylycz MJ, Puelles L, Jones AR. 2014. A high-resolution spatiotemporal atlas of gene expression of the developing mouse brain. Neuron 83:309-23.
